# Correspondence: Oncogenic MYC persistently upregulates the molecular clock component REV-ERBα

**DOI:** 10.1038/ncomms14862

**Published:** 2017-03-23

**Authors:** Brian J. Altman, Annie L. Hsieh, Arvin M. Gouw, Chi V. Dang

**Affiliations:** 1 Abramson Family Cancer Research Institute, Perelman School of Medicine, University of Pennsylvania, Philadelphia, Pennsylvania 19104, USA; 2 Abramson Cancer Center, Perelman School of Medicine, University of Pennsylvania, Philadelphia, Pennsylvania 19104, USA; 3 Division of Hematology-Oncology, Department of Medicine, Perelman School of Medicine, University of Pennsylvania, Philadelphia, Pennsylvania 19104, USA

Our report that MYC disrupts the circadian clock^1^ was corroborated by Shostak *et al*.^2^ However, in contrast to our findings that MYC induces *NR1D1* (REV-ERBα)^1^, Shostak *et al*.^2^ reported that overexpressed MYC in U2OS cells downregulated REV-ERBα and suppressed BMAL1 expression and the circadian clock through a MIZ1-dependent mechanism. They speculated that this discrepancy arose due to our documenting REV-ERBα expression at only a single-time point after MYC induction. Here, we present data in U2OS, other cancer cell lines, and a MYC-driven mouse model of liver cancer that MYC and N-MYC induce persistent upregulation of REV-ERBα, which we thus believe to be a recurrent phenomenon downstream of oncogenic MYC. Furthermore, we note from both studies that REV-ERBα and MYC/MIZ1 may in fact cooperate in repressing BMAL1, pointing to a full consistency between the mechanisms revealed in either study.

Considering a number of experimental factors, we first authenticated our U2OS MYC-ER (oestrogen receptor Tamoxifen Mutant) BMAL1::Luc cells using a detailed 16 STR (short-terminal repeat) comparison^3^ (Supplementary Tables 1 and 2 and Supplementary Note 1). We next studied whether MYC induction of REV-ERBα in U2OS and other cell lines was time-dependent. We treated U2OS MYC-ER with 4-hydroxytamoxifen (4OHT) or ethanol control for 24 h, synchronized the cells with dexamethasone, and collected mRNA and protein every 4 h. In two separate time-series experiments, we observed that MYC induction led to persistent upregulation of REV-ERBα (*NR1D1*) mRNA, upregulation of *PER2* and downregulation of *ARNTL* (BMAL1) (Fig. 1a, Supplementary Fig. 1a). MYC also upregulated REV-ERBα protein (Fig. 1b, quantitated in Fig. 1c), which was elevated at nearly every time point assessed.

Given that we employed a MYC-ER system in U2OS cells whereas Shostak *et al*.^2^ utilized a MYC TET-ON model, we asked whether persistent REV-ERBα upregulation downstream of MYC was unique to MYC-ER, but we did not address the potential experimental artifacts associated with tetracycline^4^. We used two cell models with tetracycline-inducible MYC expression: mouse hepatocellular carcinoma cells derived from a tumour with conditional MYC TET-OFF expression (‘mHCC 3–4'), and the MYC TET-OFF P493-6 human B cell line^1^,^5^,^6^,^7^. Notably, both mHCC 3–4 and p493-6 are normally cultured with elevated MYC protein which is then suppressed by tetracycline, and thus represent persistent models of MYC activation. In mHCC 3-4 cells, we observed that high MYC was associated with induced *Nr1d1* (Rev-erbα) and *Per2* mRNA, while *Arntl* (Bmal1) was suppressed at later time points in high-MYC expressing cells (Supplementary Fig. 1b). Elevated MYC was also associated with increased protein expression of Rev-erbα and Cry1 (which we previously showed was induced by MYC at the mRNA level^1^) and decreased Bmal1 (Supplementary Fig. 1c). P493-6 cells do not express detectable levels of BMAL1 mRNA, and thus have no discernible circadian oscillation. Nonetheless, after 2 h of serum shock^8^, high-MYC expression in P493-6 cells was associated with persistently elevated REV-ERBα and *PER2* expression (Supplementary Fig. 1d). P493-6 cells can also be induced to express intermediate levels of endogenous MYC protein with β-estradiol treatment^9^. In a time-series experiment conducted similarly to Supplementary Fig. 1d, we found that high MYC was associated with elevated REV-ERBα and PER2 (Supplementary Fig. 1e) as compared with intermediate MYC (Int. MYC) levels.

We previously demonstrated that N-MYC (*MYCN)*, an oncoprotein functionally related to MYC and often elevated in neuroblastoma, bound at the *NR1D1* promoter and transactivated REV-ERBα expression^1^. To examine whether N-MYC persistently induces REV-ERBα expression, we utilized two low *MYCN*-expressing neuroblastoma cell lines with inducible N-MYC, Shep N-MYC-ER^10^ and SKNAS N-MYC-ER^11^. These cells lines were treated with 4OHT or ethanol for 24 h to activate N-MYC-ER, and then synchronized with dexamethasone. In both lines, including a replicate experiment with Shep N-MYC-ER, N-MYC activation resulted in persistent upregulation of REV-ERBα and *PER2* mRNA and suppression of BMAL1 mRNA (Fig. 1d, Supplementary Fig. 2a,b). Similarly, N-MYC resulted in persistent upregulation of REV-ERBα protein and suppression of BMAL1 protein (Fig. 1e, Supplementary Fig. 2c). *PER2* protein was also upregulated by N-MYC in both cell lines. Together, these results demonstrate in a total of five distinct cell lines that MYC or N-MYC activation results in persistent upregulation of REV-ERBα.

We examined whether differences in clock synchronization and MYC induction methodology could affect REV-ERBα expression. We activated MYC for 24 h and then synchronized by stable addition of 0.1 μM dexamethasone (‘Post-Shock')^1^,^12^, while Shostak *et al*.^2^ transiently shocked cells for 20 min with 1 μM dexamethasone (‘Pre-Shock'), then washed and activated MYC after synchronization. We thus directly compared our Post-Shock with their Pre-Shock method and examined gene expression 24 and 48 h after MYC activation. With both methods, MYC significantly upregulated the mRNA expression of REV-ERBα, *PER2* and the canonical MYC target *ODC1,* while suppressing BMAL1, at both time points (Fig. 2a). We also examined protein expression, and found that with both methods MYC led to suppression of BMAL1 and early induction of PER2 (Fig. 2b). While the ‘Post-Shock' method led to increased REV-ERBα protein at both 24 and 48 h after MYC induction, with the ‘Pre-Shock' method we found REV-ERBα to already be highly elevated by dexamethasone compared to the unshocked sample (‘Post-Shock' MYC-OFF 24 h), and was not further elevated by MYC activation. Since MYC induced REV-ERBα mRNA with both methods, this non-MYC-stimulated increase in basal REV-ERBα protein may be due to increased protein production or stability. These results suggest that the different methods employed in synchronization and MYC activation both resulted in a significant induction of REV-ERBα mRNA by MYC over two time points.

We next analysed two previously published inducible MYC systems to determine whether MYC also induced REV- ERBα in these experiments. The Eilers laboratory employed a MYC-TET-ON U2OS model highly similar to Shostak *et al*.,^2^ and published RNA-seq data 30 h after MYC induction^13^. In their data, MYC significantly induced REV-ERBα, *PER2* and *ODC1* (Fig. 2c), though interestingly, BMAL1 was not suppressed at 30 h. The Amati laboratory reported RNA-seq data for a MYC TET-OFF transgenic mouse model of liver cancer^14^. In these tumours, MYC expression was associated with elevated Rev-erbα and *Odc1* and suppressed Bmal1 (Fig. 2d), while *Per2* was suppressed by MYC, consistent with previous findings that MYC can suppress *Per* expression in some models^15^.

Collectively, the data suggest that MYC induces prolonged REV-ERBα expression in two MYC-inducible models of U2OS cells, four additional cell line models of inducible MYC and N-MYC, as well as primary transgenic inducible MYC liver cancer. However, the response of other clock genes, such as *Per2,* to MYC may be context-dependent^15^. It is also notable that in contrast to Shostak *et al*.,^2^ who observed downregulation of REV-ERBα in the presence of ectopic MYC, we previously did not observe downregulation of REV-ERBα in control experiments with activation of MYC-ER Δ106-143 (ref. 1), which lacks full transactivation function but otherwise should retain the ability to interact with MIZ1. Nonetheless, double knockdown of both REV-ERB genes in our study partially rescued circadian oscillation of BMAL1::Luc in U2OS, suggesting that other mechanisms such as MIZ1 may also play a role in MYC-dependent suppression of circadian oscillation (illustrated in Supplementary Fig. 3, a model of MYC disruption of circadian rhythm by multiple and likely overlapping mechanisms). In our comparative study reported here, we cannot explain the discrepancy between our data and those of Shostak *et al*.^2^ based on methodologic differences. The body of evidence we present here suggests that MYC induction of REV-ERBα is both persistent and recurrent across many inducible MYC model systems.

## Methods

### Cell culture

U2OS BMAL1::Luc MYC-ER (oestrogen receptor Tamoxifen Mutant) was derived by stable expression of pBabe-Zeo MYC-ER in U2OS BMAL1::Luc cells^12^, and subsequent selection and culture with 100 μg ml^−1^ Zeocin (Life Technologies, Grand Island, NY, USA)^1^. Murine hepatocellular carcinoma cell line (mHCC) 3–4, a primary culture tumour cell line, was derived by ring cloning of a liver tumour from the LAP-tTA/tet-OFF cMYC conditional transgenic mouse liver cancer model^6^,^7^. U2OS, mHCC 3–4, Shep N-MYC-ER^10^ and SKNAS N-MYC-ER^11^ were cultured in Dulbecco's Modified Eagle's Medium (DMEM, Mediatech, Manassas, VA, USA) containing glucose at 25 mM and glutamine at 4 mM. Media was supplemented with 10% fetal bovine serum (FBS, HyClone, Logan, UT, USA or Life Technologies) and 1X Penicillin/Streptomycin (Mediatech). mHCC cells were additionally supplemented with 2 mM glutamine (Mediatech), 1 mM sodium pyruvate (Mediatech) and 1X MEM non-essential amino acids (Life Technologies). P493-6 cells^5^,^9^ were cultured in RPMI-1640 medium (Mediatech) with 10% FBS (HyClone or Life Technologies) and 1X Penicillin/Streptomycin (Mediatech). All cell culture was conducted in a 5% CO_2_ humidified atmosphere. For collecting cells, cells were washed one time in PBS (Life Technologies), removed from the plate with Trypsin-EDTA 0.25% (Life Technologies), suspended in media, spun down and then processed as indicated.

To activate MYC-ER or N-MYC-ER, U2OS, SHEP and SKNAS cells were treated with 500 nM 4-hydroxytamoxifen (Sigma, St Louis, MO, USA) or ethanol control. To suppress MYC expression, mHCC 3-4 cells were treated with 20 ng ml^−1^ tetracycline (Sigma) or media control for 24 h, and P493-6 cells were treated with 100 ng ml^−1^ tetracycline or media control for 24 h. To induce intermediate MYC expression (Int. MYC), P493-6 cells were treated+100 ng ml^−1^ tetracycline and 1 μM β-estradiol (Sigma) for 1 week, then cultured in β-estradiol±tetracycline for experiments.

U2OS BMAL1::Luc parental and MYC-ER were authenticated using the Cell Check 16 service (IDEXX Bioresearch, Columbia, MO, USA) with cell pellets that had been frozen. Authentication confirmed that the cells were free of interspecies contamination and genetically identical to a published U2OS profile using a detailed 16-STR (short tandem repeat) screen. U2OS BMAL1::Luc MYC-ER, mHCC 3-4 and p493-6 cells were tested for mycoplasma and found to be negative. SHEP N-MYC-ER and SKNAS N-MYC-ER were not tested.

### Real-time PCR and primers

mRNA was extracted using the RNEasy Plus Mini Kit (Qiagen, Gaithersburg, MD, USA) following manufacturer's instruction and then reverse-transcribed to cDNA by using TaqMan Reverse Transcription Reagents (Life Technologies). cDNA was used as template for quantitative real-time PCR (RT-PCR) with specific human or mouse primers. All RT-PCRs in this work were performed using the ViiA 7 Real-time PCR system (Life Technologies). Relative mRNA expression levels were normalized to *β2M* and analysed using comparative delta-delta CT method. RT-PCR primers and sources of these primers are listed in Supplementary Table 3, in the Supplementary Information section.

### Circadian time-series sample collection

**Figure 1:**
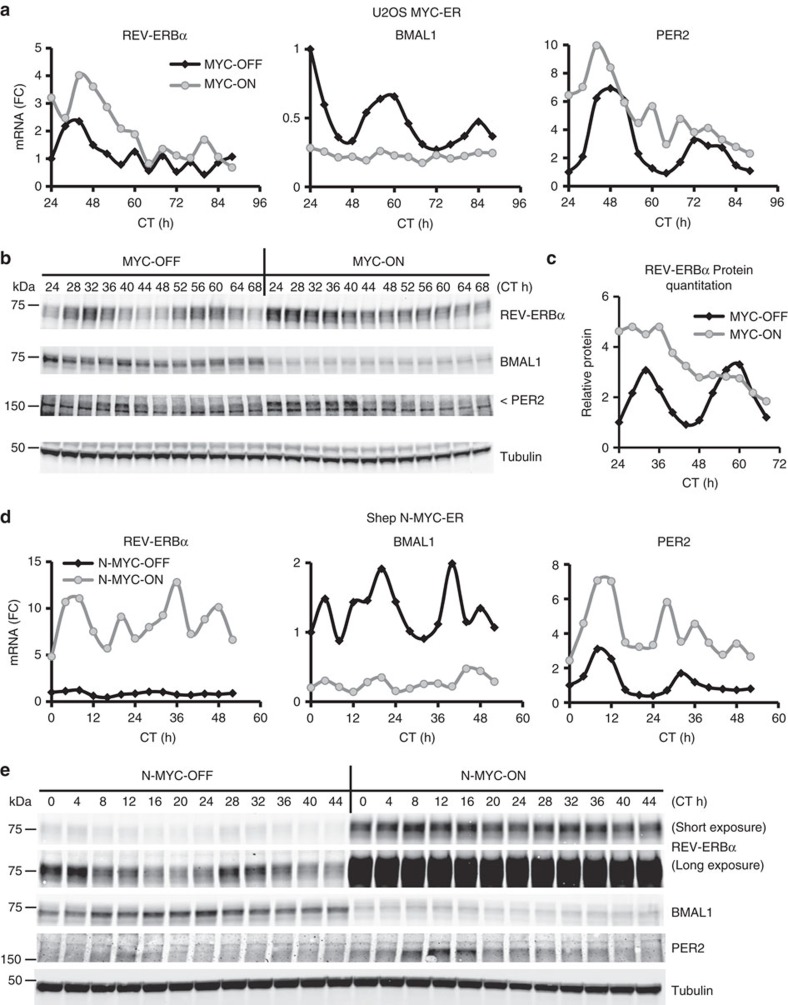
MYC and N-MYC induce persistent elevation of REV-ERBα mRNA and protein. (**a**,**b**) U2OS BMAL1::Luc cells expressing MYC-ER were cultured±4OHT (with ethanol serving as a loading control for all experiments) for 24 h, then changed to ‘lumicycle' media (see Methods)±4OHT and+0.1 μM dexamethasone. (**a**) mRNA was collected at the indicated time points after synchronization, and endogenous REV-ERBα (*NR1D1*), BMAL1 (*ARNTL*), and PER2 were determined by RT-PCR, normalized to β2M. mRNA (FC)=fold change. (**b**) Lysates were collected at the indicated time points after synchronization and processed for protein expression of REV-ERBα, BMAL1, and PER2 (indicated by <). Tubulin serves as a loading control. Portions of a and b were previously published^1^, but instead labelled as 0–48 h. (**c**) REV-ERBα protein from (**b**) was quantitated using Image Studio software (Licor, Lincoln, NE, USA), normalized to the relevant Tubulin and plotted over time. (**d**,**e**) SHEP N-MYC-ER expressing cells^10^ were cultured±4OHT for 24 h, then 0.1 μM dexamethasone was added. (**d**) mRNA was collected at the indicated time points after synchronization, and endogenous REV-ERBα (*NR1D1*), BMAL1 (*ARNTL*) and PER2, were determined by RT-PCR, normalized to β2M. (**e**) Lysates were collected at the indicated time points after synchronization and processed for protein expression of REV-ERBα (‘short' and ‘long' indicate exposure time), BMAL1 and PER2. Tubulin serves as a loading control. For all panels, CT (cell time) indicate time of collection after synchronization. For immunoblots, molecular weights are noted in kDa. Portions of **a**–**c** were previously published^1^ and are reprinted with permission from Elsevier.

All circadian time-series experiments were performed in the media described above, with U2OS, SHEP, SKNAS and mHCC 3–4 cells synchronized by addition of 0.1 μM dexamethasone (Sigma) to growth media before start of collection. As an exception, for Fig. 1a–c, U2OS MYC-ER cells were switched to ‘lumicycle media' containing dexamethasone at the start of the experiment: phenol red-free DMEM (Sigma) containing 5% FBS (Hyclone or Life Technologies), 25 mM D-glucose (Sigma), 35 mg l^−1^ sodium bicarbonate (Thermo Fisher, Grand Island, NY), 10 mM HEPES (Thermo Fisher) and Pen/Strep (Mediatech). P493-6 cells were ‘serum shocked' in 50% FBS+growth media for 2 h, then replated in normal growth media at the start of time-series collection^8^. All time-series experiments were performed as a ‘split-timecourse': 24 h before to the start of the experiment, half the cells were synchronized with 0.1 μM dexamethasone (dex, Sigma), and at the start of the experiment, the other half of the cells were synchronized with dexamethasone. At each time point, two plates were collected representing 0 and 24 h +dex, 4 and 28 and so on, to arrive at a 52 h time series. For all time-series experiments, CT denotes circadian time. mRNA and protein were processed and analysed as described above.

### Comparison of synchronization methods

To compare synchronization methods between labs, we employed ‘Pre-Shock'^1^,^12^: U2OS MYC-ER cells were first treated±4OHT for 24 h, then some cells were collected for protein and mRNA 24 h later, and others were synchronized with 0.1 μM dexamethasone and collected at 48 h±4OHT. For the method employed by Shostak *et al*.^2^ (‘Post-Shock'), we treated U2OS MYC-ER cells with 1 μM dexamethasone for 20 min, washed once in PBS, then cultured the cells in normal medium±4OHT. Cells were collected for mRNA and protein 24 and 48 h later.

### Immunoblots and immunoblot quantitation

Cells were lysed for at least 20 min in M-PER Mammalian Protein Extraction Reagent (Thermo Fisher) supplemented with protease inhibitor cocktail (BD Biosciences, San Jose, CA, or Promega, Madison, WI, USA) and phosphatase inhibitors II and III (Sigma). Lysates were centrifuged at 16,000*g* for 10 min at 4 °C, and supernatants were saved for quantitation and analysis. Protein content was quantified using the Bio-Rad DC assay kit (Bio-Rad, Hercules, CA, USA), with BSA serving as a reference (Thermo Fisher). Proteins were separated by SDS-PAGE using Criterion pre-cast gradient gels (Bio-Rad). Molecular weight was determined by comparison with Precision-Plus Dual Xtra protein standards (Bio-Rad). Primary antibodies used include: rabbit anti-REV-ERBα (1:1,000, #13418S, Cell Signaling, Danvers, MA, USA); rabbit anti-BMAL1 (1:1,000, #14020S, Cell Signaling); rabbit anti-PER2 (1:1,000, #20359-1-AP, Proteintech, Chicago, IL, USA); rabbit anti-c-MYC (1:10,000, #ab32072, Abcam, Cambridge, MA, USA); rabbit anti-CRY1 (1:1,000, #ab104736, Abcam); and mouse anti-α-Tubulin (1:10,000, #CP06, EMD Millipore, Billerica, MA, USA). Secondary antibodies used include: Alexa-Flour 680 goat anti-rabbit IgG (1:8,000, #A21109, Life Technologies, Grand Island, NY); Alexa-Flour 790 goat anti-mouse IgG (1:8,000, #A11357, Life Technologies); and IRDye 800CW Goat Anti-Mouse IgG (1:10,000, #926-32210, Licor, Lincoln, NE). Immunoblots were imaged with the Odyssey CLx infrared imaging system (Licor) and uniformly contrasted.

Immunoblots were quantitated using the Licor Image Studio software. For each band, median background intensity with a 3 pixel border width was automatically subtracted. Each REV-ERBα band value was normalized against the applicable Tubulin band, and then all the quantitated bands were further normalized by arbitrarily setting the MYC-OFF CT 24 value to 1 and dividing all the others by this value.

Raw, uncropped images of all immunoblots are presented in Supplementary Figs 4–8, along with molecular weight markers for each blot.

### Statistical analysis

For all single-time point mRNA expression data with biological replicates, error bars represent s.d., and *P<0.05 by Student's *t*-test from three experiments. For publically available data, *P<0.05 as previously described^13^,^14^.

### Data availability

For U2OS MYC TET-ON RNA-Seq, publically available data was used from Walz *et al*.^13^ (GEO accession # GSE44672, sample ID # GSM1231609). Data were supplied as log fold-change and were linearized for this work. For mouse hepatocellular carcinoma MYC TET-OFF samples, publically available data was used from Kress *et al*.^14^, supplied in a Supplementary Figure to their work. Data were supplied as log fold-change and were linearized for this work. All other relevant data are available from the authors.

## Additional information

**How to cite this article:** Altman, B. J. *et al*. Correspondence: Oncogenic MYC persistently upregulates the molecular clock component REV-ERBα. *Nat. Commun.* **8,** 14862 doi: 10.1038/ncomms14862 (2017).

**Publisher's note**: Springer Nature remains neutral with regard to jurisdictional claims in published maps and institutional affiliations.

## Supplementary Material

Supplementary Information Supplementary Figures, Supplementary Tables, Supplementary Notes, and Supplementary References

## Figures and Tables

**Figure 2:**
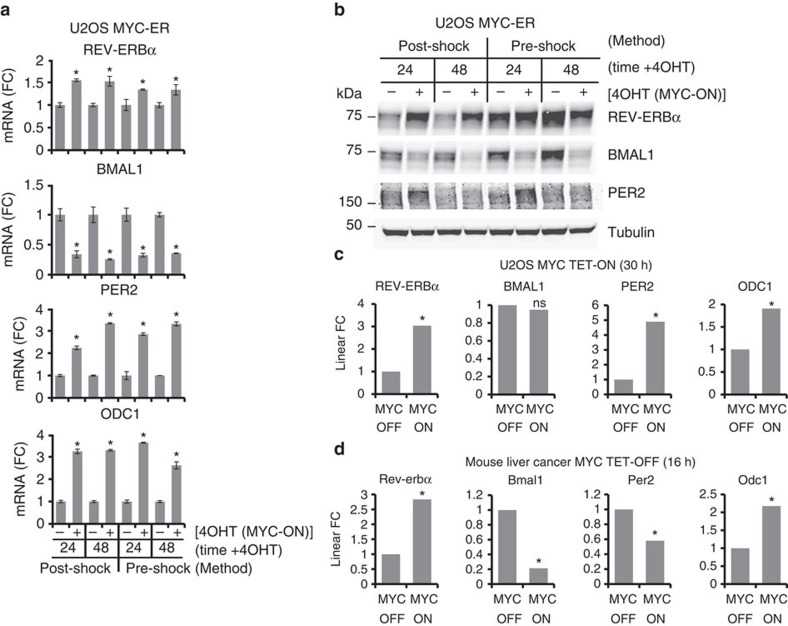
REV-ERBα mRNA is induced by MYC regardless of synchronization method and in multiple cell line and tumor models. (**a**,**b**) MYC was induced in U2OS MYC-ER cells that were synchronized with dexamethasone in two different schemes. ‘Post-Shock'^1^: as in Fig. 1, cells were treated±4 OHT (with ethanol serving as a loading control for all experiments) for 24 h. The ‘24' h sample was collected, then 0.1 μM dexamethasone was added to media and the ‘48' h sample was collected 24 h later. ‘Pre-Shock'^2^: 1 μM dexamethasone was added for 20 min, then cells were washed once in PBS and fresh media was added±4OHT. Cells were collected at the indicated times after media change. (**a**) mRNA was collected at the indicated times, and endogenous REV-ERBα (*NR1D1*), BMAL1 (*ARNTL*), *PER2*, and *ODC1* were determined by RT-PCR, normalized to β2M. mRNA (FC)=fold change. Data are averages of biological triplicates with error bars representing s.d., and *P<0.05 by Student's *t*-test of 4 OHT (MYC-ON) samples relative to ethanol (MYC-OFF) samples at each time point. (**b**) Samples from (**a**) were processed for protein expression of REV-ERBα, BMAL1 and PER2. Tubulin serves as a loading control. Molecular weights are noted in kDa. (**c**) Previously published RNA-Seq data^13^ from U2OS cells expressing exogenous MYC under the control of a TET-ON system and treated±1 μg ml^−1^ doxycycline for 30 h. REV-ERBα (*NR1D1*), BMAL1 (*ARNTL*), *PER2* and *ODC1* were determined. Data are presented as linear fold change (FC) and represent biological triplicates, and *P<0.05 of MYC-ON samples relative to MYC-OFF samples as previously described^13^; NS, not significant. (**d**) Previously published RNA-seq data^14^ from liver tumors driven by a MYC-TET-OFF system. Mice were fed water or doxycycline for 16 h to turn off MYC. Rev-erbα (*Nr1d1*), Bmal1 (*Arntl*), *Per2* and *Odc1* were determined. Data are presented as linear FC and represent biological duplicates, and *P<0.05 of MYC-ON samples relative to MYC-OFF samples as previously described^14^.
