# Supplementary material for: Correspondence: Oncogenic MYC persistently upregulates the molecular clock component REV-ERBα
Source: Nat Commun. 2017 Mar 23;8:14862. doi: 10.1038/ncomms14862 (PMC5376640; doi:10.1038/ncomms14862)
Supplement: Supplementary Information — Supplementary Figures, Supplementary Tables, Supplementary Notes, and Supplementary References [file ncomms14862-s1.pdf]

Supplementary Information

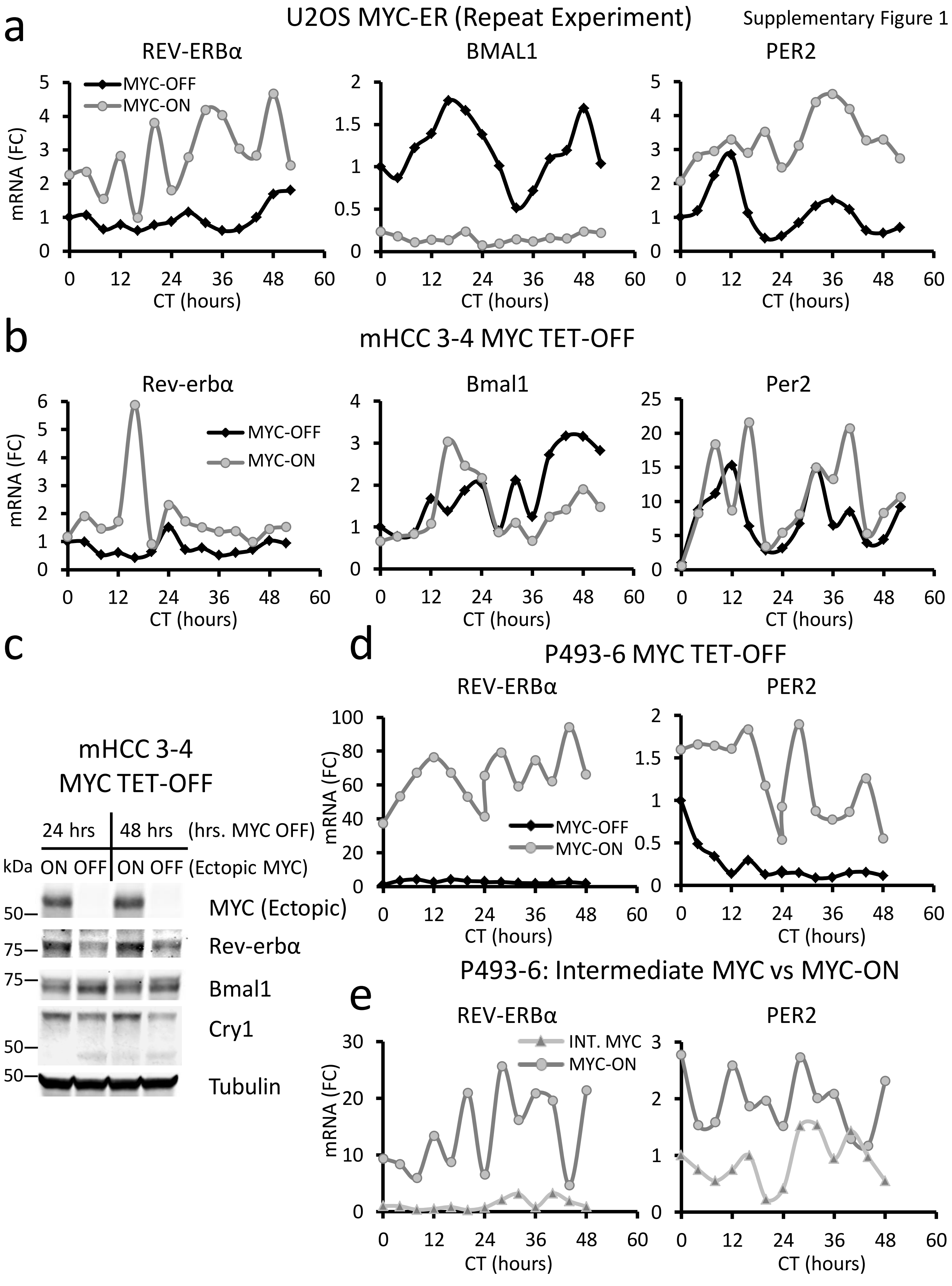

Supplementary Figure Legends

Supplementary Figure 1: **MYC promotes persistent upregulation of REV-ERBα in multiple inducible human and mouse cell line models. a**. In a replicate experiment similar to Figure 1 a-b, U2OS BMAL1::Luc cells expressing MYC-ER^{TM} [1] were cultured ± 4OHT for 24 hours, then 0.1 µM dexamethasone was added. mRNA was collected at the indicated timepoints after synchronization, and endogenous REV-ERBα (*NR1D1*), BMAL1 (*ARNTL*), and PER2 were determined by RT-PCR, normalized to β2M. **b.** Mouse hepatocellular carcinoma 'mHCC 3-4' [1,2] cells were cultured ± 20ng/ml tetracycline (tet) for 24 hours to suppress MYC expression, then 0.1 µM dexamethasone was added. mRNA was collected at the indicated timepoints after synchronization, and endogenous Rev-erbα (*Nr1d1*), Bmal1 (*Arntl*), and Per2 were determined by RT-PCR, normalized to β2M. **c.** mHCC 3-4 cells were cultured ± 20ng/ml tet for 24 or 48 hours to suppress ectopic MYC, then lysates were collected and processed for protein expression of MYC (human), Rev-erbα, Bmal1, Cry1, and Tubulin. Molecular weights are noted in kDa (kiloDaltons). **d.** Human p493-6 cells [3] were cultured ± 100ng/ml tetracycline (tet) for 24 hours to suppress MYC expression. Cells were then 'shocked' with media + 50% serum for 2 hours, then re-cultured in normal growth media ± tet. mRNA was collected at the indicated timepoints after serum shock, and endogenous REV-ERBα (*NR1D1*) and *PER2* were determined by RT-PCR, normalized to β2M. **e.** Human p493-6 cells were cultured ± tet and 1 µM β-estradiol [4] for 1 week to habituate to growth with intermediate levels of MYC ('Int. MYC'). Cells were then 'shocked' with media + 50% serum for 2 hours, then re-cultured in normal growth media ± tet and β-estradiol. mRNA was collected at the indicated timepoints after serum shock, and endogenous REV-ERBα (*NR1D1*) and *PER2* were determined by RT-PCR, normalized to β2M. mRNA (FC) = Fold Change. For all panels, CT (cell time) indicate time of collection after synchronization or serum shock. Portions of panel **b** were previously published [1] and are reprinted with permission from Elsevier.

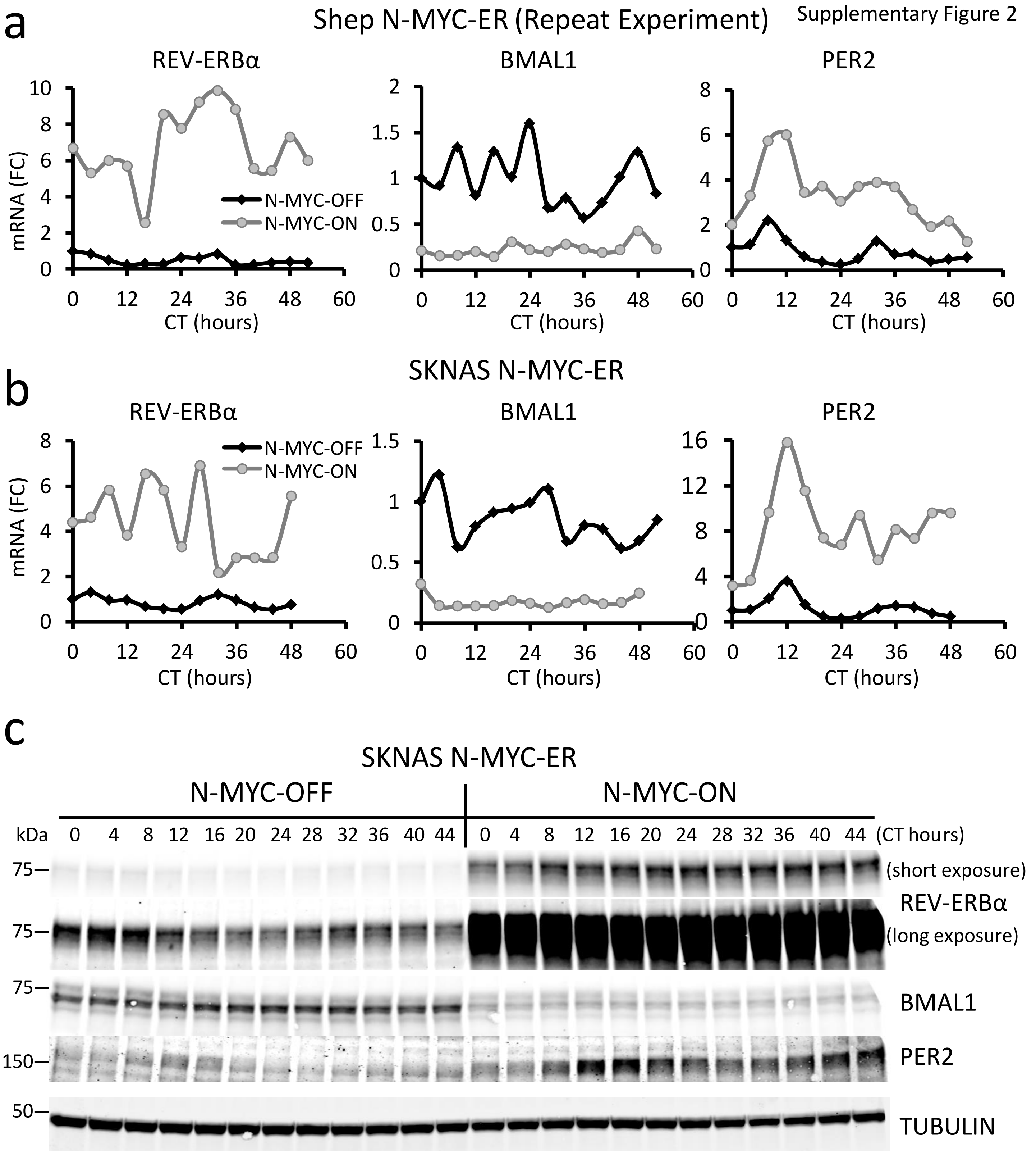
a
Shep N-MYC-ER (Repeat Experiment)
REV-ERBα
BMAL1
PER2
mRNA (FC)
N-MYC-OFF
N-MYC-ON
CT (hours)
b
SKNAS N-MYC-ER
REV-ERBα
BMAL1
PER2
mRNA (FC)
N-MYC-OFF
N-MYC-ON
CT (hours)
c
SKNAS N-MYC-ER
N-MYC-OFF
N-MYC-ON
kDa
0 4 8 12 16 20 24 28 32 36 40 44
0 4 8 12 16 20 24 28 32 36 40 44 (CT hours)
75
(short exposure)
REV-ERBα
75
(long exposure)
75
BMAL1
150
PER2
50
TUBULIN

Supplementary Figure 2: **N-MYC promotes persistent upregulation of REV-ERBα in multiple inducible neuroblastoma cell line models. a.** In a replicate experiment of Figure 1d, SHEP N-MYC-ER expressing cells [5] were cultured ± 4OHT for 24 hours, then 0.1 µM dexamethasone was added. mRNA was collected at the indicated timepoints after synchronization, and endogenous REV-ERBα (*NR1D1*), BMAL1 (*ARNTL*), and *PER2* were determined by RT-PCR, normalized to β2M. mRNA (FC) = Fold Change. **b,c.** SKNAS N-MYC-ER expressing cells [6] were cultured ± 4OHT for 24 hours, then 0.1 µM dexamethasone was added. (**b**) mRNA was collected at the indicated timepoints after synchronization, and endogenous REV-ERBα (*NR1D1*), BMAL1 (*ARNTL*), and *PER2* were determined by RT-PCR, normalized to β2M. (**c**) Lysates were collected at the indicated timepoints after synchronization and processed for protein expression of REV-ERBα ('short' and 'long' exposure time), BMAL1, and PER2. Molecular weights are noted in kDa (kiloDaltons). For all panels, CT (cell time) indicate time of collection after synchronization. Portions of panel **b** were previously published [1] and are reprinted with permission from Elsevier.

Supplementary Figure 3

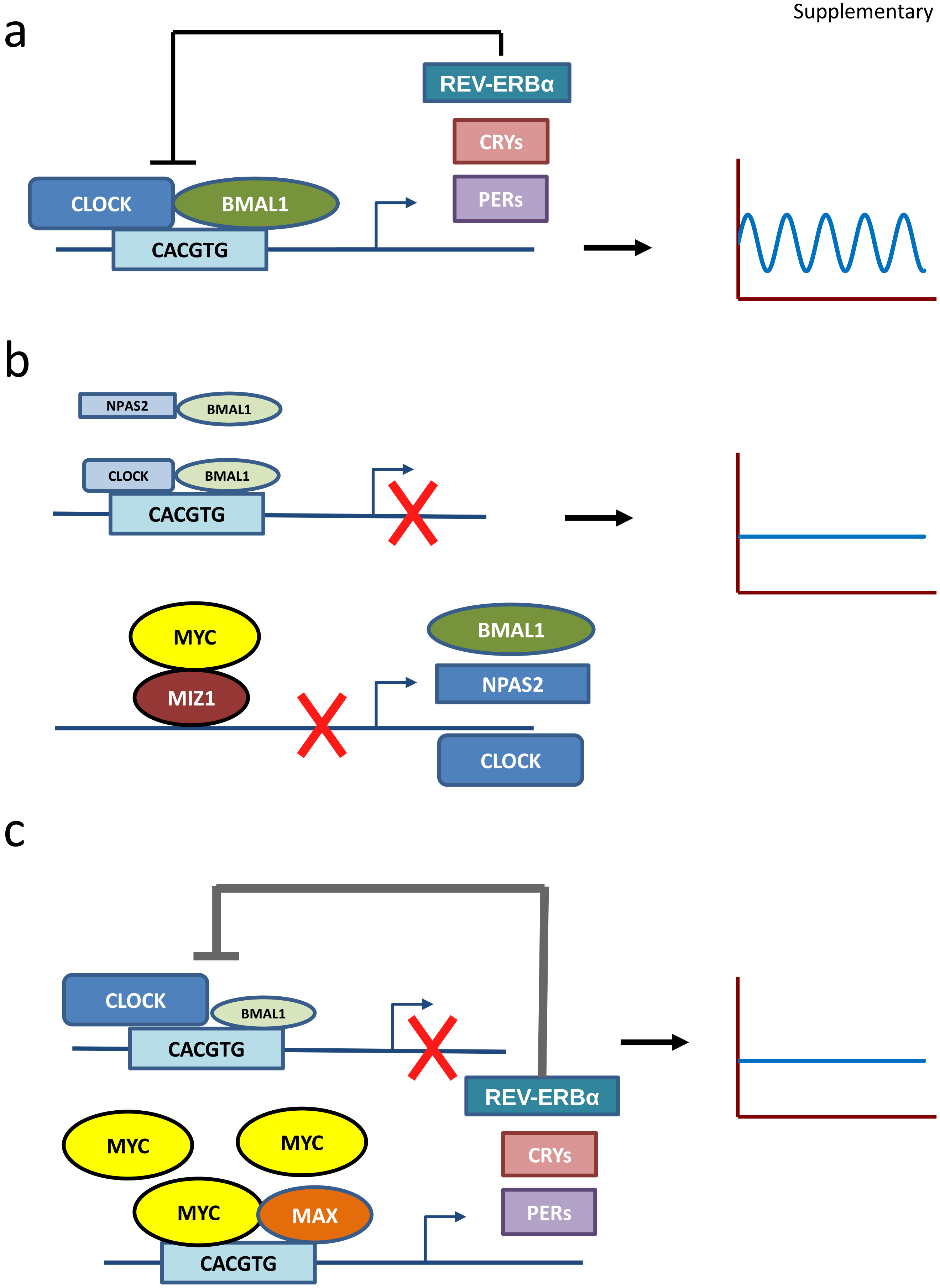

Supplementary Figure 3: **MYC disrupts circadian rhythm through two complementary pathways. a.** Simplified model of the molecular circadian clock. CLOCK-BMAL1 control the expression of several negative regulatory factors such as PER, CRY, and REV-ERBα, which feed back to control CLOCK-BMAL1 activity and BMAL1 expression, leading to roughly 24 hour oscillations. **b.** As previously described by Shostak and colleagues [7], the transcription factor MIZ1 positively regulates BMAL1, CLOCK, and the CLOCK analogue NPAS2. Ectopic MYC binds to and inhibits MIZ1, thus depriving cells of BMAL1, CLOCK, and NPAS2 and leading to disruption of circadian oscillation. **c.** As we previously described [1], ectopic MYC can upregulate many negative regulatory elements of the CLOCK by direct promoter binding and expression. In particular, MYC upregulation of REV-ERBα leads to reduced BMAL1 expression and disruption of circadian oscillation.

Raw uncropped immunoblots for Figure 1b (U2OS MYC-ER circadian time-series ± MYC activation)

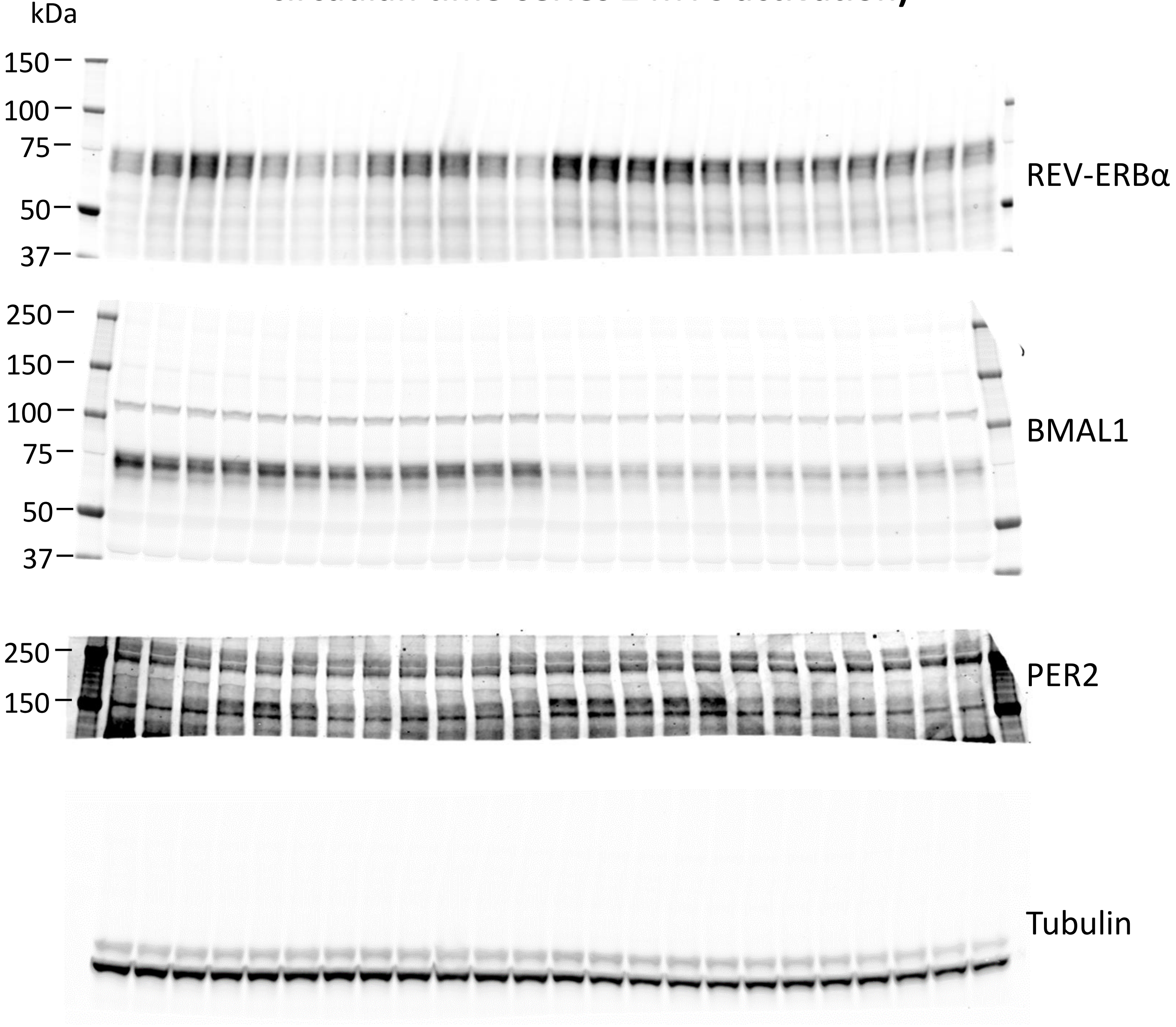

Supplementary Figure 4: **Raw uncropped immunoblots from Figure 1b.** U2OS MYC-ER circadian time-series ± MYC activation. Molecular weight markers shown are from the Bio-Rad Protein Plus Dual Xtra Standards.

Raw uncropped immunoblots for Figure 1d (SHEP N-MYC-ER circadian time-series ± N-MYC activation)

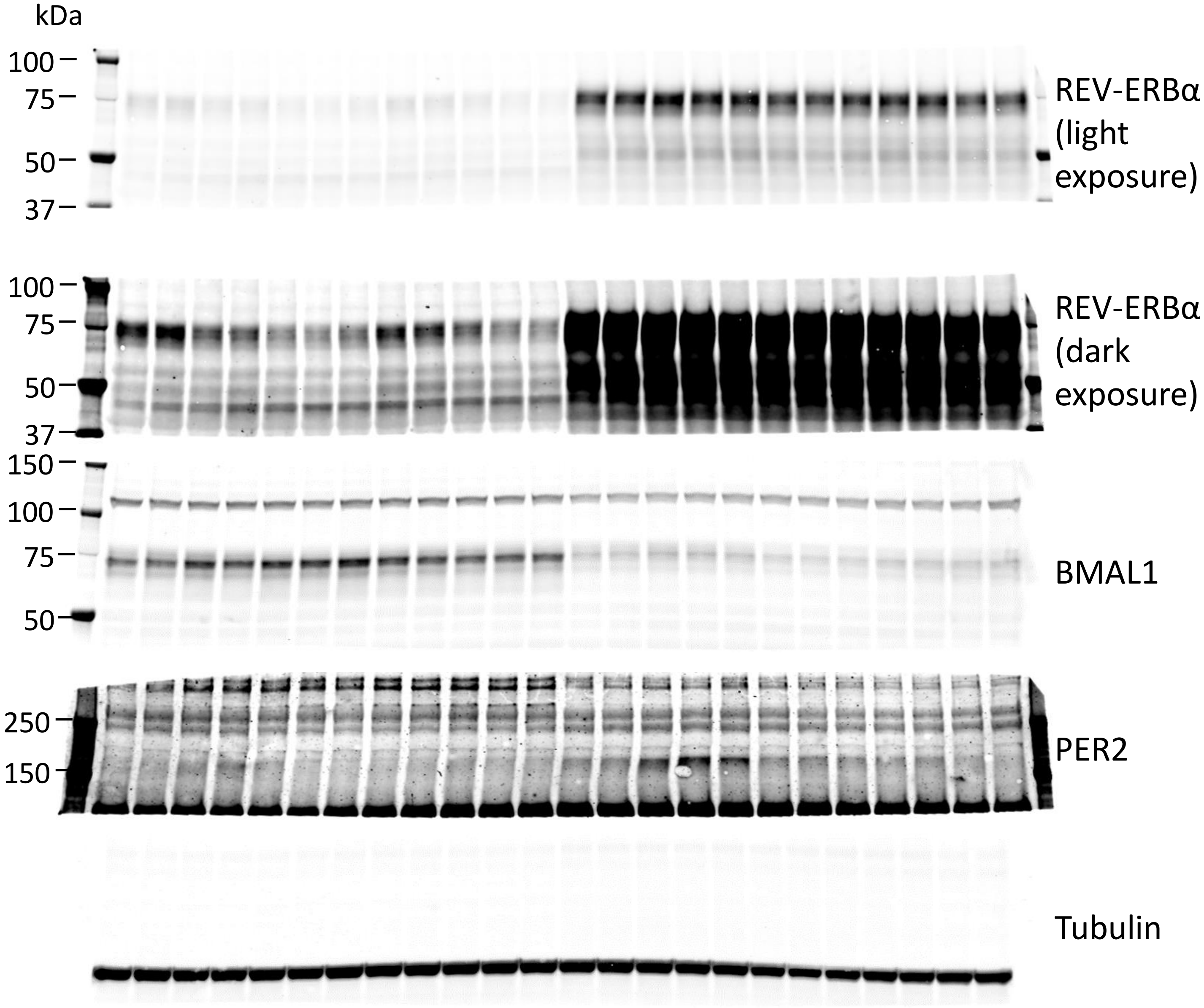

Supplementary Figure 5: **Raw uncropped immunoblots from Figure 1d.** SHEP N-MYC-ER circadian time-series ± N-MYC activation. Molecular weight markers shown are from the Bio-Rad Protein Plus Dual Xtra Standards.

Raw uncropped immunoblots for Figure 2b (U2OS MYC-ER comparison of circadian synchronization methods ± MYC activation

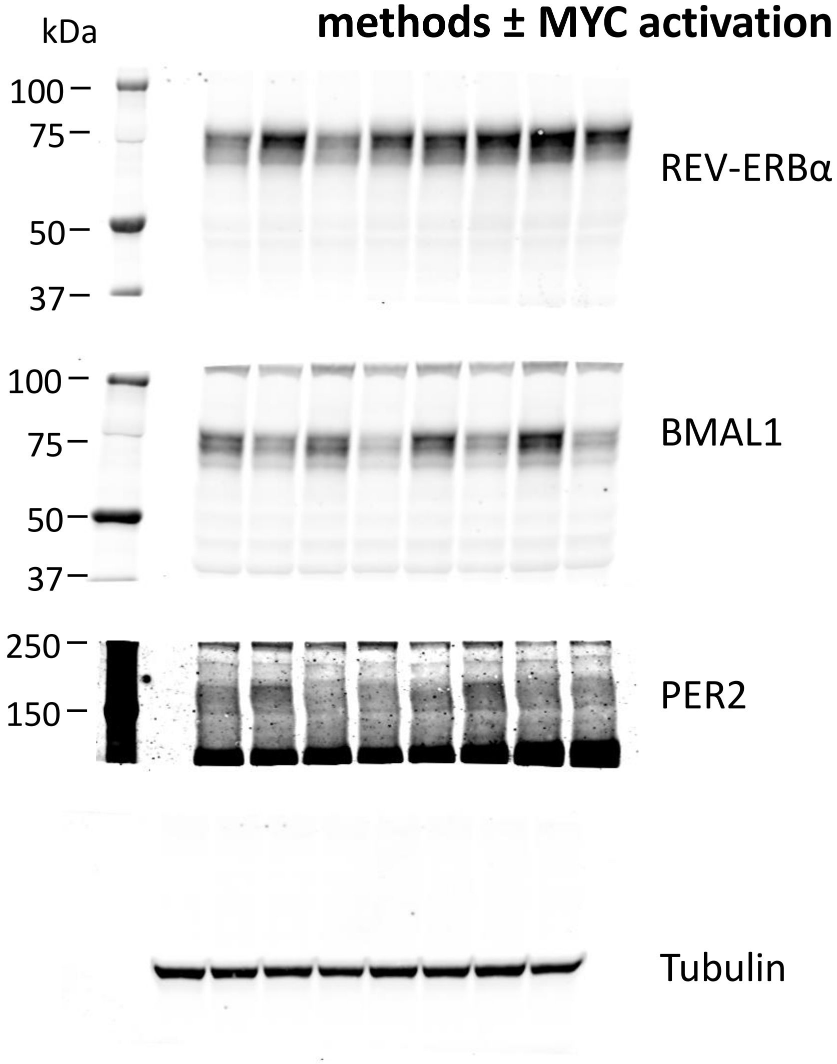

Supplementary Figure 6: **Raw uncropped immunoblots from Figure 2b.** U2OS MYC-ER comparison of circadian synchronization methods ± MYC activation. Molecular weight markers shown are from the Bio-Rad Protein Plus Dual Xtra Standards.

Raw uncropped immunoblots for Supplementary Figure 1c (mHCC 3-4 cells ± ectopic TET-OFF MYC protein at two timepoints)

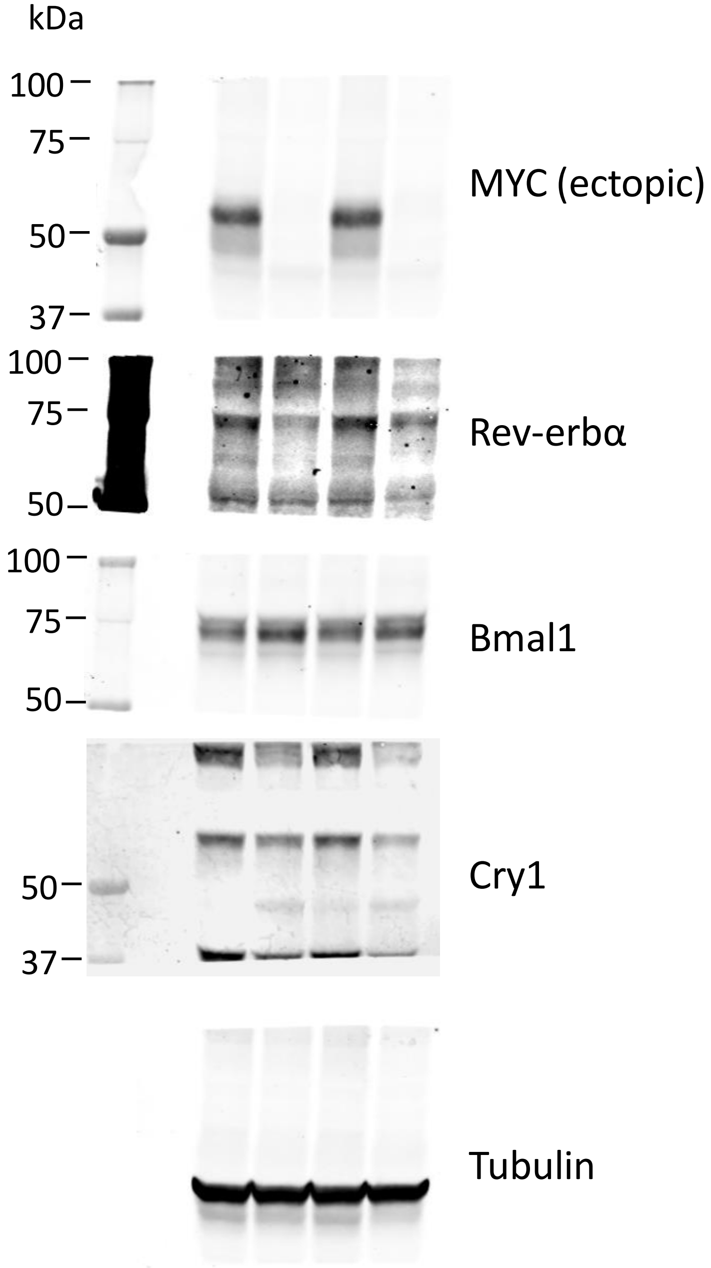

Supplementary Figure 7: **Raw uncropped immunoblots from Supplementary Figure 1c.** mHCC 3-4 cells ± ectopic TET-OFF MYC protein at two timepoints. Molecular weight markers shown are from the Bio-Rad Protein Plus Dual Xtra Standards.

Raw uncropped immunoblots for Supplementary Data Figure 2c (SKNAS N-MYC-ER circadian time-series ± N-MYC activation)

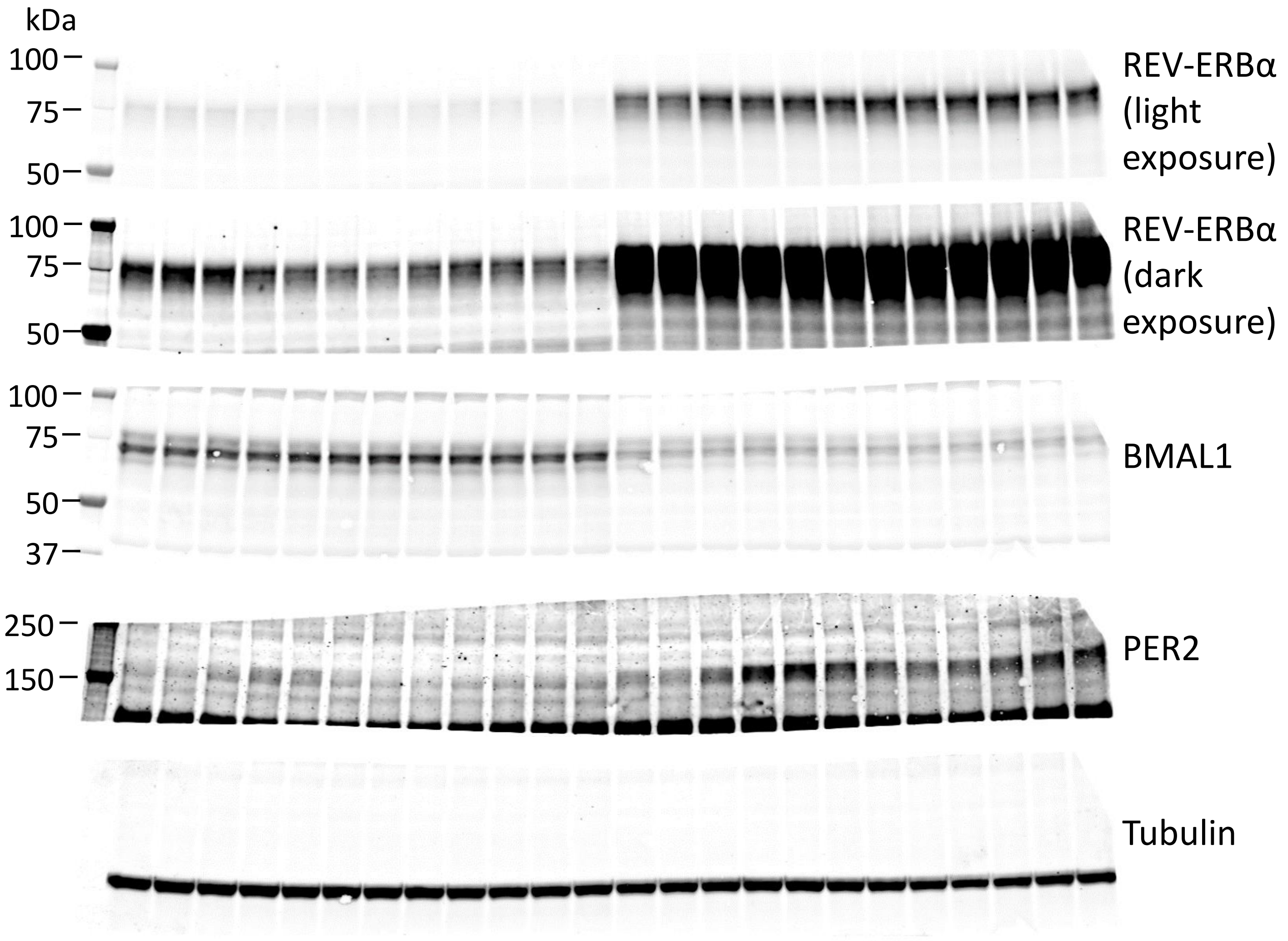

Supplementary Figure 8: **Raw uncropped immunoblots from Supplementary Figure 2c.** SKNAS N-MYC-ER circadian time-series ± N-MYC activation. Molecular weight markers shown are from the Bio-Rad Protein Plus Dual Xtra Standards.

Supplementary Table 1

Accession #: 16383-2016 IDs: B, D

Services Performed:
CellCheck 16 (16-Marker STR Profile and Inter-species Contamination Test)

ID	Client ID	Cell Line	Species	Strain/Breed
B	B	U2OS Bmal::Luc Polyclonal	Human	ATCC HTB-96
D	D	U2OS Bmal::Luc cMYC-ER	Human	ATCC HTB-96

Summary: Cell Check results are provided in the data results section for each sample. For human samples, an identity matching score above 80% indicates the sample is consistent with the cell line of origin. For human samples with less than an 80% matching score, please see individual comments for these samples in the detail section.

CellCheck
Species-specific PCR Evaluation

Species	Sample B	Sample D
mouse	-	-
rat	-	-
human	+	+
Chinese hamster	-	-
African green monkey	-	-

Supplementary Table 2

Marker Name	B			D		
	Sample Results	U-2 OS (ATCC# HTB-96)	U-2 OS (Nature 520:307-311, 2015) 131871	Sample Results	U-2 OS (ATCC# HTB-96)	U-2 OS (Nature 520:307-311, 2015) 131871
AMEL	X	X	X	X	X	X
CSF1PO	12, 13	13	13	12, 13	13	13
D13S317	13	13	13	13	13	13
D16S539	11, 12	11, 12	11, 12	11, 12	11, 12	11, 12
D18S51	12, 14	NA	14	12, 14	NA	14
D21S11	31	NA	31	31	NA	31
D3S1358	16	NA	16	16	NA	16
D5S818	8, 11	11	11	8, 11	11	11
D7S820	11, 12	11, 12	12	11, 12	11, 12	12
D8S1179	12, 14, 15	NA	12, 14	12, 14, 15	NA	12, 14
FGA	20	NA	20	20	NA	20
Penta_D	9	NA	9	9	NA	9
Penta_E	10, 13	NA	10, 13	10, 13	NA	10, 13
TH01	6, 9.3	6, 9.3	6, 9.3	6, 9.3	6, 9.3	6, 9.3
TPOX	11, 12	11, 12	11, 12	11, 12	11, 12	11, 12
vWA	14, 18, 19	14, 18	14, 18	14, 18, 19	14, 18	14, 18
Identity Match	>80%			>80%		

Supplementary Note 1

The sample was confirmed to be of human origin and no mammalian interspecies contamination was detected. The alleles for 16 different markers were determined and the results were compared to the alleles reported for the cell line and to the parental cell line. The 9 marker genetic profile established by ATCC (NA indicates markers for which ATCC has not established an allele size) and the 16 marker genetic profile published in Nature are provided for comparison. The sample has an extra allele at markers D8S1179 and vWA, but is otherwise identical to the parental cell line profile. While the sample has minor genetic differences compared to the established reference profiles and the profile of the parental cell line, the identity matching score is above 80% confirming the sample is consistent with the cell line of origin.

Supplementary Tables 1-2 and Supplementary Note 1: **U2OS MYC-ER^{TM} are authentic U2OS cells and free of interspecies contamination.** Cell line authentication using U2OS BMAL1::Luc cells [8] (labeled 'B') and U2OS BMAL1::Luc MYC-ER^{TM} [1] (labeled 'D') was performed at IDEXX Bioresearch (Columbia, MO, USA) with cell pellets that had been frozen. **Supplementary Table 1.** Both cell lines were found to be free of interspecies contamination from other mammalian cell lines. **Supplementary Table 2.** A PCR screen was performed using 16 short tandem repeats (STRs), and compared to both an ATCC (Manassas, VA, USA) reference sample as well as a more detailed published 16-STR profile [9]. Both U2OS cell lines had identical STR profiles and were over 80% identical to the published 16-STR profile. **Supplementary Note 1.** Statement from IDEXX Bioresearch stating that U2OS BMAL1::Luc MYC-ER^{TM} cells are over 80% identical to a published U2OS profile, and are therefore authentic U2OS cells.

Supplementary Table 3

Gene Name	Sequence or Product Number	Source
	**Human Primers**	
REV-ERBα (*NR1D1*)	TGGACTCCAACAACAACACAG, GATGGTGGGAAGTAGGTGGG	Primer Bank [10] ID#300116298c1
BMAL1 (*ARNTL*)	Hs00154147_m1	Taqman Gene Expression Assay
PER2	GGATGCCCGCCAGAGTCCAGAT, TGTCCACTTTCGAAGACTGGTCGC	Primer Blast
ODC1	CTT CGT GCA GGC AAT CTCT, TCT CTT CAA ATT TAA GTT TCA CAT CCT	IDT (Hs.PT.51.22750281.gs)
β2M	GGCCGAGATGTCTCGCTCCG, TGGAGTACGCTGGATAGCCTCC	Primer Blast
	**Mouse Primers**	
Rev-erbα (*Nr1d1*)	Mm00520708_m1	Taqman Gene Expression Assay
Bmal1 (*Arntl*)	Mm00500226_m1	Taqman Gene Expression Assay
Per2	Mm00478113_m1	Taqman Gene Expression Assay
Odc1	Mm.PT.53a.23589427	Taqman Gene Expression Assay
β2m	ACCGGCCTGTATGCTATCCAGAAA, GGTGAATTCAGTGTGAGCCAGGAT	Previously Published [11]

Supplementary Table 3. **Sequences of qPCR Primers Used.** Forward and reverse primer sequences, and sources of these sequences, are provided.

Supplementary References

1. Altman, B.J., *et al.* MYC Disrupts the Circadian Clock and Metabolism in Cancer Cells. *Cell Metab* **22**, 1009-1019 (2015).
2. Shachaf, C.M., *et al.* MYC inactivation uncovers pluripotent differentiation and tumour dormancy in hepatocellular cancer. *Nature* **431**, 1112-1117 (2004).
3. Schuhmacher, M., *et al.* Control of cell growth by c-Myc in the absence of cell division. *Curr Biol* **9**, 1255-1258 (1999).
4. Yustein, J.T., *et al.* Induction of ectopic Myc target gene JAG2 augments hypoxic growth and tumorigenesis in a human B-cell model. *Proc Natl Acad Sci U S A* **107**, 3534-3539 (2010).
5. Ushmorov, A., *et al.* N-myc augments death and attenuates protective effects of Bcl-2 in trophically stressed neuroblastoma cells. *Oncogene* **27**, 3424-3434 (2008).
6. Valentijn, L.J., *et al.* Inhibition of a new differentiation pathway in neuroblastoma by copy number defects of N-myc, Cdc42, and nm23 genes. *Cancer Res* **65**, 3136-3145 (2005).
7. Shostak, A., *et al.* MYC/MIZ1-dependent gene repression inversely coordinates the circadian clock with cell cycle and proliferation. *Nature communications* **7**, 11807 (2016).
8. Baggs, J.E., *et al.* Network features of the mammalian circadian clock. *PLoS Biol* **7**, e52 (2009).
9. Yu, M., *et al.* A resource for cell line authentication, annotation and quality control. *Nature* **520**, 307-311 (2015).
10. Spandidos, A., Wang, X., Wang, H. & Seed, B. PrimerBank: a resource of human and mouse PCR primer pairs for gene expression detection and quantification. *Nucleic Acids Res* **38**, D792-799 (2010).
11. Altman, B.J., *et al.* Autophagy provides nutrients but can lead to Chop-dependent induction of Bim to sensitize growth factor-deprived cells to apoptosis. *Mol Biol Cell* **20**, 1180-1191 (2009).
